# Basophils in pruritic skin diseases

**DOI:** 10.3389/fimmu.2023.1213138

**Published:** 2023-07-03

**Authors:** Daniela Wiebe, Maren M. Limberg, Natalie Gray, Ulrike Raap

**Affiliations:** ^1^ Division of Experimental Allergy and Immunodermatology, School of Medicine and Health Sciences, Carl von Ossietzky University Oldenburg, Oldenburg, Germany; ^2^ Division of Anatomy, School of Medicine and Health Sciences, Carl von Ossietzky University Oldenburg, Oldenburg, Germany; ^3^ Research Center for Neurosensory Science, Carl von Ossietzky University Oldenburg, Oldenburg, Germany; ^4^ University Clinic of Dermatology and Allergy, University of Oldenburg, Oldenburg, Germany

**Keywords:** basophils, IL-31, atopic dermatitis, neuro-immune interaction, pruritus

## Abstract

Basophils are rare cells in the peripheral blood which have the capability to infiltrate into the skin. Invasion of basophils has been detected in pruritic skin diseases, including atopic dermatitis, bullous pemphigoid, chronic spontaneous urticaria and contact dermatitis. In the skin, basophils are important players of the inflammatory immune response, as they release Th2 cytokines, including interleukin (IL)-4 and IL-13, subsequently inducing the early activation of T-cells. Further, basophils release a multitude of mediators, such as histamine and IL-31, which both play an important role in the initiation of the pruritic response *via* activation of sensory nerves. Chronic pruritus significantly affects the quality of life and the working capability of patients, though its mechanisms are not fully elucidated yet. Since basophils and neurons share many receptors and channels, bidirectional interaction mechanisms, which drive the sensation of itch, are highlighted in this review.

## Introduction

**Figure 1:**
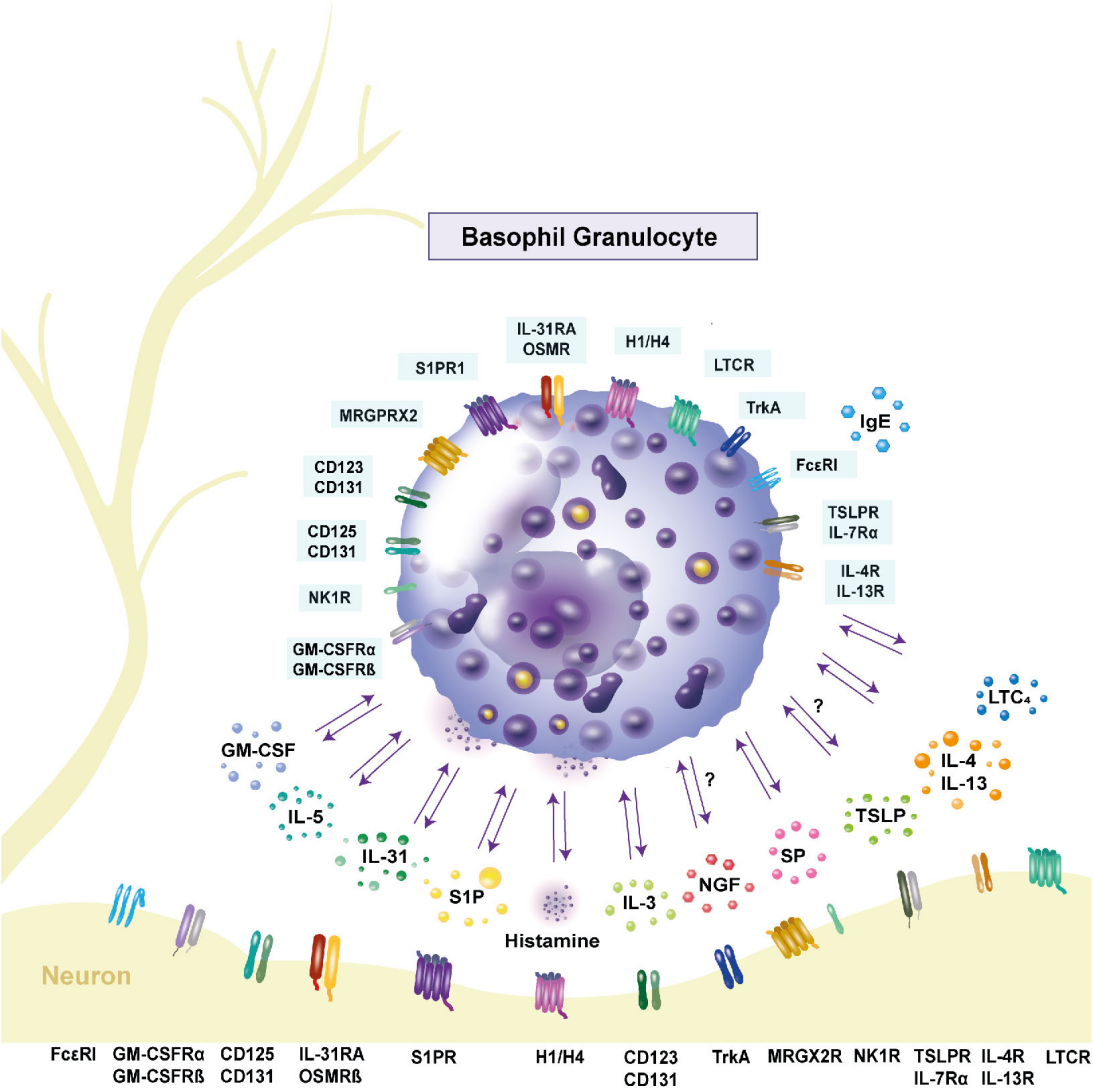
Expression of receptors and release of cytokines in human basophils. Basophils interact with other immune cells and neurons through inflammatory mediators and receptor expressions. Interleukin (IL)-31, as well as its receptor complex consisting of the IL-31 receptor A and the oncostatin M receptor β, are expressed by basophils and contribute to pruritus. Stimulation with IL-31 leads to the secretion of the pro-inflammatory cytokines IL-4 and IL-13. Their respective receptors are IL-4R and IL-13R. Basophils express the high-affinity receptor FcϵRI. Upon crosslinking of the receptor with IgE, histamine is released, mediating itch. The hormone receptors are present on the cell surface, with the histamine 4 receptor being the most highly expressed. Activation of the neurokinin 1 receptor through substance P (SP) also causes histamine release. Basophils can be primed by IL-5, IL-3 and granulocyte macrophage-colony stimulating factor (GM-CSF). The respective receptors are CD125 and CD131 for IL-5, CD123 and CD131 for IL-3 and the GM-CSF receptor consists of GM-CSFRα and GM-CSFRβ. Activation of these receptors leads to increased histamine release. Another priming factor is nerve-growth factor, which binds to the tyrosine kinase A receptor on the cell surface. Basophils express the Mas-related G protein-coupled receptor X2 (MRGPRX2), which is part of the signaling cascade in inflammation and serves as a receptor for SP. Another pruritogen is thymic stromal lymphopoietin (TSLP), which binds to the TSLP receptor complex consisting of TSLP receptor and IL-7 receptor α and is proposed to cause itch. Whether basophils respond to TSLP is controversial. The lipid mediator sphingosine-1-phosphate (S1P) is stored in granules and its receptor S1P receptor 1 is expressed on the cell surface. It is proposed to have an anti-inflammatory effect on basophils. The leukotriene C4 (LTC4) is released by basophils and its receptor cysteinyl leukotriene receptor (LTCR) is expressed by basophils. GM-CSF: granulocyte macrophage-colony stimulating factor; GM-CSFRα: GM-CSF receptor α; GM-CSFRβ: GM-CSF receptor β; H1/H4: histamine 1/4 receptor; IL: interleukin; IL-4R: IL-4 receptor; IL-5R: IL-5 receptor; IL-7RA: IL-7 receptor α; IL-13R: IL13 receptor; IL-31RA: IL-31 receptor A; LTC4: leukotriene C4; LTCR: cysteinyl leukotriene receptor; MRGPRX2: Mas-related G protein-coupled receptor X2; NK1R: neurokinin 1 receptor; OSMRβ: oncostatin M receptor β; trkA: tyrosine kinase receptor A; TSLP: thymic stromal lymphopoietin; TSLPR: TSLP receptor; SP: substance P; S1P: sphingosine-1-phosphate; S1PR1: S1P receptor 1.

Basophil granulocytes are named due to their affinity to basic dyes (1). The diameter of basophils is 10 - 14 µm (2) and basophils are the least abundant type of granulocytes in human blood, where they comprise less than 1% of all leucocytes (1). After differentiation from hematopoietic stem cells in the bone marrow, fully matured basophils enter the blood stream (2). Basophils do not proliferate (3) and have a short lifespan of 60 - 70 h in mice (4). In humans, lifespans of up to 11 days have been reported (5). During helminth elimination, basophils are involved in protective mechanisms and also play a significant role in enhancing inflammation (6). Basophils are an important early source of Th2-type cytokines such as interleukin (IL)-4 and IL-13 in inflammation (**Figure 1**) (7). Moreover, basophils release the pruritic cytokine IL-31, and express its receptor complex consisting of the IL-31 receptor A (IL-31RA), and the oncostatin M receptor β (OSMRβ) (**Figure 1**, **Table 1**) (21). Stimulating basophils with IL-31 induces basophil chemotaxis and promotes the secretion of Th2 cytokines (21). Another itch mediator is histamine. The pruritogen is released after activation of the high-affinity IgE receptor FcϵRI (**Figure 1**) (33). A specific characteristic of human basophils is the potentiation of mediator release after stimulation with priming factors. In the pathogenesis of inflammatory diseases, enhancing factors, such as IL-3, nerve growth factor (NGF), IL-5 and granulocyte macrophage-colony stimulating factor (GM-CSF), modulate the functional activity of basophils. IL-3 is the most potent activator of basophils and also promotes basophil differentiation (35). Its receptor α-chain CD123 is expressed by basophils (**Figure 1**) (13–17). Another priming agent for basophils is the neurotrophin NGF, which induces the release of histamine and the synthesis of leukotriene C4 (LTC4) after stimulation with agonists (**Figure 1**) (36). NGF has similar effects on basophils as IL-5 and GM-CSF (36). While IL-5 belongs to the group of Th2 cytokines (37), GM-CSF is a monomeric glycoprotein that is present at sites of tissue inflammation (38). Both are produced by basophils and promote inflammation (39). Activation of basophils is associated with upregulation of the cell surface markers CD13, CD45, CD63, CD203c (40), and CD69, for which increased expression is mostly observed after stimulation with IL-3 (41). A method to assess human basophil activation is to determine changes in the amount of these surface proteins. The most reliable activation markers are CD63 and CD203c (40). CD63 is a membrane protein, that is associated with histamine containing granules. After anaphylactic degranulation (42, 43), CD63 is translocated to the cell surface of activated basophils as a result of histamine release (43). The ectoenzyme CD203c (pyrophosphatase/phosphodiesterase) is weakly expressed on resting basophils (44). Whereas CD63 externalization is closely related to basophil degranulation (44). Upon activation, CD203c, which is not associated with mediator release, is upregulated rapidly (43). Basophil infiltration has been observed in atopic dermatitis (AD), bullous pemphigoid (BP), chronic spontaneous urticaria (CSU) and contact dermatitis (7), all of which are pruritic inflammatory skin diseases. The mechanism how basophils are recruited into the skin remains to be fully elucidated. It is assumed that basophils are attracted by a variety of mediators present in the skin, i.e. the chemokines, CCL2, CCL5, CCL11, CXCL12, and prostaglandin D2 (45). Basophils express the respective receptors, CCR4 for CCL2 and CCL5, CCR3 for CCL11, CXCR4 for CXCL12 and chemoattractant receptor-homologous molecule expressed on Th2 cells (CRTH2) for prostaglandin D2 (45, 46). CCL11 is produced by dermal fibroblasts and CRTH2 is elevated in AD (46). Other potential chemoattractants of basophils are thymic stromal lymphopoietin (TSLP), IL-3, IL-31, histamine, substance P (SP) and sphingosine-1-phosphate (S1P). TSLP and IL-3 cause the upregulation of CXCR4 and thereby lead to infiltration of basophils into the skin (47). The pruritogen IL-31 has been shown to induce chemotaxis in basophils *in vitro* (21). Upon histamine release from mast cells, murine basophils are recruited to the site of allergen exposure in nasal tissue (48). SP has also been shown to chemoattract basophils, resulting in the infiltration of basophils into the skin of healthy individuals (49). Recently, it was shown that in healthy donors, basophils migrate towards S1P which was observed in an *in vitro* study, while in AD patients a chemorepulsive effect was detected (31). It has however, so far not been described if basophils, that migrated into the skin, return to the blood or travel to draining lymph nodes (45, 50). Pruritus elicits the desire to scratch the skin and is categorized into acute and chronic pruritus. Chronic itch, by definition, lasts longer than 6 weeks, and strongly impairs patients’ quality of life. Although its complete mechanism has yet to be elucidated, complex crosstalk between the stratum corneum, keratinocytes, immune cells, and nerve fibers (**Figure 1**) plays an important role in the initiation and maintenance of pruritus. Itch can originate in the skin or have neuropathic, psychogenic or systemic causes (51). Histamine, IL-31, SP, LTC4, IL-4, IL-13, NGF, brain-derived neurotrophic factor (BDNF), and TSLP, which all are released by or affect basophils (**Figure 1**), have been reported to cause itch (7, 12, 21, 52–54) and are described in the chapter “Basophils and neuro-immune interactions”. Current therapies for itch target different receptors on basophils, such as IL-31RA, neurokinin 1 receptor (NK1R), tropomyosin-receptor kinase A (trkA), or released mediators, i.e. IL-13. The monoclonal IL-31RA antibody nemolizumab binds to IL-31RA and thereby interrupts IL-31 itch signaling in basophils. A trial from Japan in which nemolizumab was administered, found improvements in pruritus and quality of life, leading to the approval of the drug for AD (55). NK1R is expressed in basophils and its antagonists inhibit pruritic signaling and decrease itch in patients. However, the inhibitors are not licensed for use (56). In mice, treatment with signal transducer and activator of transcription 6 (STAT6) inhibitors led to decreased scratching. IL-13 targets STAT6, inducing pruritus (57). Janus kinase (JAK) inhibitors interrupt the JAK-STAT signaling pathway. This disruption, which occurs after treatment with JAK inhibitor upadacitinib, leads to improvement of pruritus in patients (58). Application of the trkA inhibitor CT327 resulted in a significant decrease of pruritus in psoriasis patients (59). The role of basophils as important effector cells in different inflammatory skin diseases and their involvement in pruritus, are described in the following chapters.

**Table 1:** Shared receptors of basophils and neurons with their respective ligand.

Shared receptor	Ligand	References
GM-CSFRα/β	GM-CSF	(8, 9)
H1/H4	Histamine	(10, 11)
IL-4R	IL-4	(12)
IL-3R	IL-3	(13–18)
IL-5R	IL-5	(19, 20)
IL-13R	IL-13	(12)
IL-31 receptor complex	IL-31	(21, 22)
LTCR	LTC4	(23)
MRGPRX2	SP	(24, 25)
NK1R	SP	(12, 26, 27)
trkA	NGF	(28, 29)
TSLP receptor complex	TSLP	(12, 30)
S1PR1	S1P	(31, 32)
FcϵRI	IgE	(33, 34)

GM-CSF, granulocyte macrophage-colony stimulating factor; GM-CSFRα/β, GM-CSF receptor α and β; H1/H4, histamine 1/4 receptor; IL, interleukin; IL-3R: IL-3 receptor; IL-4R: IL-4 receptor; IL-5R, IL-5 receptor; IL-7RA, IL-7 receptor α; IL-13R, IL13 receptor; IL-31RA, IL-31 receptor A; LTC4, leukotriene C4; LTCR, cysteinyl leukotriene receptor; MRGPRX2, Mas-related G protein-coupled receptor X2; NK1R, neurokinin 1 receptor; OSMRβ, oncostatin M receptor β; trkA, tyrosine kinase receptor A; TSLP, thymic stromal lymphopoietin; TSLPR, TSLP receptor; SP, substance P; S1P, sphingosine-1-phosphate; S1PR1, S1P receptor 1.

## Atopic dermatitis

Atopic dermatitis (AD) is an inflammatory skin disease, associated with recurrent dry skin, and the main bothersome symptom, itch (60). In patients with AD, infiltration of basophils into the skin and peripheral blood has been observed, although not in as high numbers as in other skin diseases (46, 61). In one study, significantly less basophil numbers could be detected in peripheral blood of AD patients than in healthy controls (62). Interestingly, increased basophil count is suggested to be a potential causal risk factor for AD (63). Basophils were found to exhibit increased externalization of the activation markers CD63 and CD203c in AD patients (61). This indicates possible involvement of basophils in the pathogenesis of AD. Basophils can be primed by NGF (**Figure 1**), which is produced by a variety of cells, such as keratinocytes (53), eosinophils (64), T cells (65), and mast cells (66). NGF has been shown to be either increased (53), or significantly decreased in AD patients, correlating with disease severity when compared to healthy subjects (67). In lesional skin of subjects with AD, the number of NGF positive nerve fibers is increased (68). Whether basophils are a source of NGF, has yet to be elucidated. In the epidermis, the lipid mediator sphingosine-1-phosphate (S1P) plays an important role regarding structure, lipid signaling and the regulation of keratinocytes. Our group recently discovered that isolated basophils of atopic patients exhibited decreased S1PR1 expression, and possessed intracellular S1P in isolated basophils (31). Furthermore, in the stratum corneum of AD patients, the lipid is decreased, which might alleviate colonization with *Staphylococcus aureus* (69). The lipid, as well as mRNA expression of the S1P receptors (S1PR) S1PR1, S1PR2, S1PR3 and S1PR4, have been observed in human basophils (**Figure 1**) (31). The presence of S1PR1 was also confirmed at the cell surface (31) (**Table 1**). S1PR1, S1PR2 and S1PR4 have been detected in the brain (32), indicating another point of neuro-immune crosstalk. Due to the inhibiting effect of the lipid mediator on chemotaxis, S1P is proposed to have an anti-inflammatory effect on basophils (31). In both mice and humans, significant upregulation of FcϵRI on basophils during AD has been observed, indicating that IgE might also be an important factor in pruritus (70). The pro-inflammatory effect of basophils in AD might be reduced by treatment with dupilumab. The monoclonal IgG4 antibody, which binds to IL-4Rα, showed success in reducing symptoms, such as itch, of AD patients (71). Since the antibody binds to IL-4Rα, the assumption arises, that the cytokines which contribute to the disease are partially derived from basophils (71). Aside from their pro-inflammatory properties, basophils can aid in the resolution of AD. The expansion of M2-like macrophages was promoted by murine basophils, as well as epidermal repair (72), which additionally affirms the role of basophils in AD.

## Bullous pemphigoid

Bullous pemphigoid (BP) is a blistering skin disease, that most commonly occurs in elderly people and only rarely affects adolescents or children. An autoimmune reaction against the hemidesmosomal proteins BP180 and BP230 leads to the formation of blisters (73). A case study showed that basophil infiltration took place in early- as well as late-stage lesions (74). The twofold involvement of basophils in BP was shown by Kimura et al. (75). During the early stage of BP, basophil infiltration was correlated with eosinophil infiltration. Cell-to-cell contact was observed, indicating that Th2 immunity is promoted by eosinophils and basophils (75). A case study detected the colocalization of basophils and eosinophils in urticarial plaques (74). The presence of basophils was also demonstrated, as well as eosinophils, underneath the subepidermal cleft during the late-stage of BP (74). Basophils in BP were shown to be present with a high density, similar to that observed in urticaria, but higher than that in AD (46), and increased compared to skin healthy controls (76). Circulating basophils from untreated BP patients were stimulated with BP180, resulting in significantly higher histamine release than those basophils of treated BP patients or healthy controls (77). This suggests an important role for basophils in the development of BP. The amount of anti-basement membrane zone antibodies was positively correlated with IgE serum levels (78). Treatment with the anti-IgE monoclonal antibody omalizumab resulted in the downregulation of FcϵRI on basophils in two cases (79). Activation of basophils was determined through measuring CD203c expression. The expression was evaluated before and after treatment with two doses of prednisolone and three sessions of plasma exchange, and found to be significantly reduced after treatment (74). These observations indicate that basophils play a role in the development of BP. In BP, itch is an important factor, which is confirmed as itch severity correlates with the increased numbers of basophils present in the blisters (76). Thus, basophils seem to play an important role in pruritus, blister development and inflammation in BP.

## Chronic spontaneous urticaria

Chronic spontaneous urticaria (CSU) presents in patients as pruritic hives, angioedema or a combination of both (80). Patients suffering from CSU often present with peripheral basopenia, where low amounts of basophils are present in the blood, probably due to the infiltration into the skin (81). An inverse correlation between disease severity and the amount of basophils in the blood has been observed (81). Moreover, significantly more infiltrating basophils are present in lesions of CSU patients than in nonatopic subjects (82). Basophil degranulation has also been observed in the skin of CSU patients. Therefore, the reactivity in CSU seems to be partially regulated by basophils (82). Substance P (SP) was shown to be positively correlated with the number of basophils in the peripheral blood of CSU patients (26). Interestingly, basophil numbers were increased in CSU patients compared to healthy controls, in contrast to findings of other studies. These basophils exhibited higher expression levels of SP, as well as its associated receptor NK1R, than those from healthy controls. When activated by its agonist, NK1R mediated up to 41% net histamine release, which is comparable to that induced by anti-IgE and the chemoattractant N-formylmethionyl-leucyl-phenylalanine (fMLP) (26). A similar effect was confirmed in mice. Blood basophil numbers increased after injection with SP. Sensitization with ovalbumin resulted in elevated basophils numbers as well as increased SP and NK1R expression on basophils (26). As itch is a significant symptom of CSU, its origin is important. One causative factor might be IL-31, which is elevated in this disease (83). Basophils have been reported to be the main source of IL-31 in skin lesions of CSU (**Figure 1**) (21). In CSU, patients can be categorized in three groups; responders, nonresponders and basopenics, depending on how much histamine is released from basophils after stimulation with anti-IgE (84). Upon application of anti-IgE, basophils of responders release high amounts of histamine and exhibit increased CD63 externalization. Nonresponders are characterized by low histamine secretion and CD63 externalization, while almost no reaction can be observed in basophils of basopenics (84). Responders, those with high histamine release, seem to suffer from CSU longer than the other groups. However, the number and size of hives, as well as the itch score were highest in basopenics (84). Another study confirmed that the duration of the disease is longer in responders. The same group of patients also reported increased itch (85). Treatment with the anti-IgE monoclonal antibody omalizumab showed a decrease of symptoms in CSU patients (86). Furthermore, the number of peripheral blood basophils increased as a result of treatment with omalizumab (87). Whether the monoclonal antibody inhibits basophil migration into the skin, or promotes the release of new basophils from the bone marrow has yet to be investigated. Basophils of CSU patients exhibited significantly higher amounts of CD63, than those of healthy controls. CD203c expression however was unchanged (88). In contrast, another study revealed no difference of activation marker levels in CSU patients in comparison with healthy subjects. However, histamine release was reported to be higher in patients with CSU than in controls (89). In CSU patients in remission, basophils were more activated, as determined through the presence of CD63 and CD203c, than in healthy control (90). This shows that basophils are crucial in the development of CSU.

## Contact dermatitis

Irritant contact dermatitis is characterized by non-allergic, pruritic skin inflammation, where basophils infiltrate into the tissue (91). In human and murine irritant contact dermatitis skin lesions, basophils were located in proximity to eosinophils, which were recruited to the site by the basophils (91). Furthermore, in mice, direct cell-to-cell contact of basophils with eosinophils seems to lead to the activation of eosinophils, enhancing the development of irritant contact dermatitis (91). Allergic contact dermatitis, however, is caused by contact with an allergen, which also induces basophil migration. Interestingly, infiltration lasts for several days, where basophils can be detected after 25 hours and then increase in number in allergic contact dermatitis (92). Basophils represent 16% of the infiltrate in allergic contact dermatitis at day 16, resulting in delayed hypersensitivity (92). In accordance with this finding, degranulation of basophils was observed to occur over 72 hours, where approx. 60% of granules were found to be at least partially depleted (93). Eosinophil infiltration occurs after basophil infiltration, indicating that basophils play a role in eosinophil recruitment in contact dermatitis (92). Thus, basophils play an important role in the aspects of cell infiltration and pruritus during the development of irritant and allergic contact dermatitis.

## Basophils and neuro-immune interaction

Interactions between the immune system and the nervous system play an important role in inflammatory skin diseases and pruritus. These neuro-immune interactions stem from intense crosstalk between neurons and immune cells, which are located in close proximity to one another. Upon allergen challenge with the irritant calcipotriol and the allergen ovalbumin, murine basophils migrate into the skin, and are consistently observed to be located in close proximity to sensory nerve fibers (**Figure 1**), indicating neuroimmune interactions (70). The initiation and maintenance of itch is characterized by many mediators expressed by basophils, including IL-31, SP, LTC4, histamine, IL-4 and IL-13. Other pruritic mediators, such as TSLP, NGF and BDNF also affect basophils. It is assumed, that basophils interact bidirectionally with neurons through cytokines and neurotrophins, as they share various channels and surface receptors (**Figure 1**, **Table 1**). While IL-31RA is present on most basophils, OSMRβ can only be found on a small subpopulation (21). IL-31RA is expressed on half of dorsal root ganglia (DRG) with a size up to 30 µM (22), and its ligand can act as a neurotrophin on DRG neurons (94). Through activation of IL-31RA (22) on peripheral nerves, itch signals are transmitted to the central nervous system (21).

The Mas-related G-protein-coupled receptor (MRGPR) X2 is expressed on human basophils (**Figure 1**, **Table 1**) (24) and DRG (25), and evokes allergic, as well as nonallergic hypersensitivity (32). In mice, the transient receptor potential ankyrin 1 (TRPA1) channel is necessary for MRGPR- and TSLP-mediated pruritus (95). Upon activation, the channel is opened and induces itch (95).

Basophils release the inflammatory mediator LTC4. Its receptor cysteinyl leukotriene receptor 2 (CysLTR2) is expressed on basophils and DRG (23).

After priming with IL-3, human basophils express the TSLP receptor, while expression of the IL-7 receptor α was not detectable (96). In contrast, mice express TSLPR and IL-7 receptor α on basophils (**Figure 1**), which together form the TSLP receptor complex (30). Stimulation of basophils with TSLP has been shown to cause histamine release, and increase intracellular IL-4 and IL-13 expression, as well as induce the upregulation of TSLPR in patients with allergic asthma (97). In contrast to this study, Guen et al. reported that basophils from healthy and allergic patients did not respond to TSLP (98). The TSLP receptor complex has also been confirmed in DRG. Observations in mice revealed TSLP secretion from basophils and activation of neurons through the cytokine (12). TSLP activates TRPA1 expressing neurons and causes itch (99). Secretion of TSLP by human basophils has not yet been investigated.

Basophils and peripheral nerve endings express the tachykinin neurotransmitter SP and its receptor NK1R (**Figure 1**) (12, 26, 27). The neuropeptide is involved in inflammation and itch (12). Furthermore, SP induces histamine release from basophils, indicating possible interactions between the nervous system and the granulocytes (26, 27), as basophils are able to communicate with neurons *via* histamine. Basophils express the histamine-1 receptor and histamine-4 receptor (H4R; **Figure 1**) (10), which have also been confirmed to be expressed in the central nervous system (11). When H4R is activated on basophils, it mediates chemotaxis. However, activation can also lead to basophil silencing, as CD63 and CD203c surface content has been observed to be suppressed and the production and release of sulfidoleukotrienes reduced (10). In mice, knockout of H4R resulted in reduced inflammation and treatment with H4R antagonists alleviated itch (12).

Secretion of IL-4 and IL-13 from basophils (**Figure 1**), indicates communication between basophils and neurons in pruritus. Their respective receptor subunits IL-4Rα and IL-13Rα are expressed in basophils, as well as in DRG (12). In a murine model, injection of IL-4 caused scratching, suggesting that IL-4 induces pruritus in mice (12).

Neurotrophins play an important role in the communication between basophils and neurons. Basophils and the central nervous system express tyrosine kinase receptor A (**Figure 1**) (100, 101), to which NGF binds. NGF is also a priming factor for basophils, demonstrating the influence of the neuronal system on basophils. To conclude, interaction between basophils and the neuro-immune system occurs through a variety of channels and mediators, highlighting the importance of basophils in neuro-immune interaction mechanisms.

## Conclusion

Basophils play a crucial role in many pruritic inflammatory skin diseases. In these conditions, basophils are among the first cells to infiltrate into the skin. At this location, basophils secrete Th2 cytokines and are drivers of the inflammation. The pruritic effect is further mediated by IL-4, IL-13, IL-31, histamine, SP, TSLP, BDNF and NGF, of which most are released by basophils. IL-31 is a key mediator in itch, its expression being increased in inflammatory and pruritic skin diseases. Basophils also recruit eosinophils to sites of inflammation in BP and CSU, further increasing the inflammation. Moreover, basophils are able to establish cell-to-cell contact with sensory neurons, and enable neuro-immune interaction through the release of inflammatory mediators, such as IL-31. Thus, basophils seem to be major drivers of inflammation and itch in diseases such as AD, BP, CSU and contact dermatitis, which was summarized in this review.

## Author contributions

Conceptualization, DW and UR. Writing—original draft preparation, DW. Writing—review and editing, ML, NG, UR. Illustration, ML. All authors contributed to the article and approved the submitted version.
